# Social processing of dynamic naturalistic social interactions

**DOI:** 10.1177/17470218251346724

**Published:** 2025-05-17

**Authors:** Katie Daughters, Simona Skripkauskaite, Kami Koldewyn

**Affiliations:** 1 Department of Psychology, University of Essex, Colchester, UK; 2 Department of Experimental Psychology, University of Oxford, Oxford, UK; 3 Department of Psychiatry, University of Oxford, Oxford, UK; 4 Department of Psychology, School of Psychology and Sport Science, Bangor University, Bangor, UK

**Keywords:** Social interaction, social attention, naturalistic, dynamic

## Abstract

Research suggests that static depictions of social interactions preferentially capture our attention compared to non-interactions. Research also suggests that motion captures attention. To date, therefore, it is unknown whether *dynamic* social interactions preferentially capture attention relative to non-interactions, over and above motion cues. The present study captured 81 participants’ eye-gaze when viewing 4-s video clips of social interactions compared to motion-matched non-interactions. We hypothesised that participants would spend more time looking at the two agents in the videos relative to the background when viewing social interactions compared to non-interactions. Results confirmed our hypothesis and demonstrated that this effect was stronger for individuals with greater empathy and lower autistic traits. These results add to the growing body of research investigating the processing of social interactions in complex, naturalistic stimuli and demonstrate that social interactions do preferentially capture attention, even when motion cues are present.

## Introduction

Humans are an inherently social species. As such, researchers have sought to understand how we make sense of and engage with our social world. This has often meant focusing on one particular facet of social perception and conducting tightly controlled laboratory experiments using simplified stimuli. There is, however, a growing demand to move away from this approach to better understand how we process more complex and naturalistic social information, and in particular, whether this processing is specialised to or different for social interaction perception.

A large body of research has investigated visual attention as an important mechanism underlying social perception. It is known that social stimuli quickly and effectively capture attention relative to non-social stimuli (End & Gamer, 2017). More recently, studies have demonstrated a visual preference for ‘interactive’ or facing human dyads and triads compared to non-facing dyads and triads (Fratino et al., 2022; Papeo & Abassi, 2019; Papeo et al., 2019; Vestner et al., 2019; Vestner, Flavell, et al., 2022). Why might individuals spend more time looking at two agents when they are interacting as opposed to when they are engaged in pursuing separate goals, particularly when scenes contain the same number of people? On the surface, scenes where agents pursue separate goals might require *more* attention and processing to parse than scenes where individuals engage in joint actions. However, as discussed in prior work (Skripkauskaite et al., 2022), social interactions constitute more than the sum of their parts; for example, they provide information on the social context and the relationship between two individuals. There may also be attentional cues common to many social interactions that capture attention preferentially because such cues are themselves spatial attentional cues. Vestner and colleagues have proposed that, because body orientation and gaze both act as spatial attentional cues (Vestner, Gray, et al., 2021), interactions where two people directly face each other may create an ‘attentional hot spot’ that captures attention (Vestner, Over, et al., 2021). They suggest that this mechanism could explain at least some of the preferential attention to social interactions, particularly as they demonstrate similar increased attention to facing pairs than non-facing pairs for objects that also cue spatial attention (e.g. lamps, arrows; Flavell et al., 2022; Vestner, Gray, et al., 2022; Vestner, Over, et al., 2022)). In the observation of social interactions ‘in the wild’, it is likely that both social context and content and general attentional cues that are not specific to social scenes will contribute to preferential attention to social interactions.

Importantly, however, recognising social interactions in the real world also relies on a diverse set of cues. Most prior work investigating attention to social interactions has used simple silhouettes/figures and manipulated ‘interactiveness’ only through facing direction, contrasting facing dyads with dyads that either face away from each other or face in the same direction. While such stimuli are tightly controlled, they do not encompass other important indicators to social interaction, such as eye-gaze or gestures. Additionally, social encounters unfold dynamically across time such that different cues (both attentional and social) may drive observer’s attention at different points in time. The ‘cognitive ethological approach’ suggests that to better grasp attention in genuine social encounters, different types of social stimuli that range in their approximation to a real social interaction need to be studied (see Kingstone, 2009; Risko et al., 2012).

A few studies have included more complex stimuli that are nearer to ‘real’. For example, Villani et al. (2015) presented participants with paintings depicting two individuals either acting independently or interacting with one another. They found that participants spent more time looking at the bodies of individuals when they were interacting and more time looking at the face when acting independently. However, this study did not include a direct comparison of attention to social versus non-social information, and thus it is not possible to ascertain the moderating impact of social interaction on this contrast; they also used paintings, not photographs depicting real life. A more recent study (Skripkauskaite et al., 2022) expanded on this by investigating visual attention to interacting and non-interacting dyads in real-life photographs. They found that participants spent more time looking at the agents compared to the background for interacting stimuli, and there was no difference for non-interacting stimuli, supporting the idea that social interactions, specifically, capture social attention.

These studies, however, have all utilised static images. Dynamic stimuli, on the other hand, not only enable the use of more ecologically valid stimuli but also incorporate additional indicators to social interaction such as actions and gestures, particularly those that are directed towards another human (Wurm & Caramazza, 2019). From much previous literature, we might expect attentional processes to differ between static and dynamic stimuli for both social and non-social reasons. Indeed, prior research demonstrates that movement quickly and effectively captures attention regardless of scene type (e.g. Atkinson et al., 2018). Introducing movement therefore provides an additional challenge to determining whether social interactions capture attention differently or preferentially as previous studies using static stimuli would suggest. There is some preliminary evidence to support this hypothesis. For instance, observation of dynamic, compared to static, interactions give rise to different neural representation patterns (Landsiedel et al., 2022). Furthermore, Chevallier et al. (2015) found that children spent more time looking at faces compared to objects during dynamic videos that depicted multiple agents and showed both social interactions and non-interactions. This analysis, however, focused on faces exclusively, not whole individuals, and did not explicitly analyse differences between interactions and non-interactions. Thus, it remains unknown whether social interactions drive visual attention even in complex dynamic scenes where general motion cues and other ‘low level’ visual features may compete for attention.

Finally, it is important to consider individual differences that may bias one’s visual attention. Individuals who are higher in trait empathy have been found to be faster to look at human figures (Villani et al., 2015) in response to static stimuli. In contrast, autistic individuals have been found to spend less time looking at human information compared to neurotypical individuals (Chevallier et al., 2015; Shi et al., 2015). Thus, although research suggests there are differences in visual attention for those with higher trait empathy and autistic traits, this is yet to be examined in naturalistic dynamic stimuli and has not been investigated with respect to social interaction perception.

The primary aim of the current study was to investigate visual attention as a potential mechanism underlying social perception of dynamic social scenes, and to assess to what extent the presence of a social interaction may moderate this effect. The secondary aim was to investigate the effect of individual differences on visual attention to observed social interactions, contrasted with visually similar non-interactions. This was examined across three hypotheses: (a) Participants will spend more time looking at the agents (vs. the background) and this will be moderated by the presence of social interactions, such that participants will spend more time looking at the agents (versus the background) in social interactions compared to non-interactions; (b) Participants higher in trait empathy will display greater visual attention in response to social interactions (compared to non-interactions) (c) Participants lower in autistic traits will display greater visual attention in response to social interactions (compared to non-interactions).

## Method

### Participants

An a priori power calculation, based on previous research (Skripkauskaite et al., 2022), determined that 70 participants would be sufficient to attain 80% power with an ⍺ = .05 to detect a three-way interaction with a large effect size (Cohen’s *f* = 0.4), however, due to anticipated data loss, the study aimed to recruit 85 participants. Eighty-one participants (19–84, *M* = 30.78, *SD* = 13.85; 45 females) took part in the in-person study at ESSEXLabs. Participants were recruited via an online booking system and received financial compensation for taking part. The data were pre-processed and analysed in accordance with pre-registration (https://aspredicted.org/blind.php?x=VGV_Y4C).^1^ Data from seven participants were removed due to true outliers (e.g. technical issues); none of the participants was excluded due to missing eye-tracking data; 15 participants had missing questionnaire data and were therefore not included in the individual difference analyses. The study was approved by the University of Essex Department of Psychology Ethics Committee (ETH2122-1116).

### Materials

#### Social perception task

**Figure 1.**
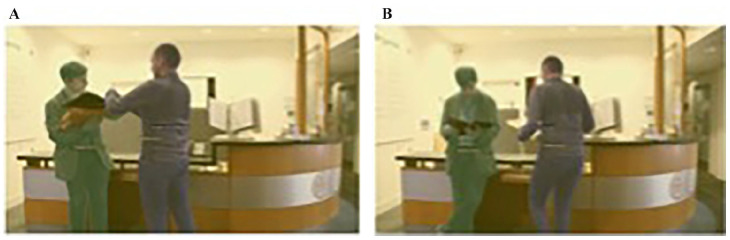
Example of AOIs created for each video. (A) Interaction video; (B) Non-interaction video. Yellow AOI represents the background AOI, the green and purple AOI represent the agent AOIs. *Note*. AOI = areas of interest.

Participants were asked to watch 60 video clips depicting various everyday scenarios. Each 4-s video clip depicted a scenario with two agents either engaging in a social interaction or acting independently (non-interaction). In total, there were 30 interaction and 30 non-interaction scenarios where each interaction scene was matched as closely as possible to a non-interaction scene on facing direction, eye-gaze and inter-personal distance such that both scenes contained the same agents and objects and agents performed similar actions (e.g. Figure 1). Matched interaction and non-interaction scenes were filmed from the same camera angle in the same setting and did not differ in overall motion energy (see Landsiedel et al., 2022 for analysis). Deliberate and careful attention was paid to matching each unique scenario *across* conditions. At the same time, these various important cues to social interaction (proximity, facing direction, etc.) were also deliberately varied *within* conditions, such that the 30 social interaction videos were as varied as possible; for some videos, facing direction was the most salient cue to interaction, while in others joint attention might have been the most salient. For example, in one scenario, two agents are initially seated next to one another at a table. Both have a mug in hand, and one also has a mobile phone. One agent gets up and moves behind the chair of the other agent (moves towards/faces the agent and gains proximity) and then continues to walk away from them (faces away from the agent, increasing distance). In the social interaction condition, there is a mutual eye-gaze at the beginning and a small nod, but then the moving agent doesn’t look back while the other glances at her phone as the agent moves behind her, briefly watches the other agent begin to walk away and then returns her gaze to her phone. In the non-interaction condition, the agent that moves does exactly the same movement but without the initial look at the other agent, there is no nod and the seated agent looks at her phone. Thus, the agents, movement, facing direction and props are all the same with only small deviations in eye-gaze and brief indicators of non-verbal communication. A sample of the stimuli are provided on the study’s OSF page (https://osf.io/dqjpr/?view_only=01bdb55be14148e4ae4121b5f64489fa).

Interaction videos were previously subjectively rated as more social, more positive and more visually interesting than non-interaction videos (Landsiedel et al., 2022). Scenarios were acted out by four different agent pairs (two female/female pairs, one male/male pair and one female/male pair) captured in eight different locations (e.g. an office, lobby waiting area, etc.).

The experimental task was programmed using iMotions (www.imotions.com), a biometric research platform that can be used to synchronise multiple psychophysical measures. This enabled eye-tracking data and stimulus presentation to be precisely coordinated. Eye-tracking data were recorded using a portable Tobii x2-120 compact eye-tracker sampling at 120 Hz with a screen resolution of 1,920 × 1,080. An I-VT fixation filter was applied, and data were sampled from both eyes to produce information on eye position and latency.

#### Questionnaires

##### Autistic traits

The Autism Quotient Short version (AQ-S; Kloosterman et al., 2011) is an adapted 28-item version of the original 50-item AQ (Baron-Cohen et al., 2001). Items relate to five subscales: social skills, mind reading/communication, restricted and repetitive behaviour, imagination and attention to detail. Participants were asked to rate to what extent they agree/disagree with each item (1 = *definitely agree*; 4 = *definitely disagree*). The measure includes reverse item scoring such that higher scores represent more autistic traits. A total score and mean scores for each subscale were calculated. The total score achieved good internal reliability (Cronbach’s ⍺ = .78).

##### Trait empathy

The Interpersonal Reactivity Index (IRI; Davis, 1983) is an established questionnaire, with items pertaining to four subscales: empathic concern, fantasy, personal distress and perspective taking. There are 28 items in total, seven for each subscale (including reverse-coded items). For each item, participants are asked to indicate on a 5-point scale to what extent the statement can be applied to them (1 = ‘does not describe me very well’; 5 = ‘describes me very well’). A total score and mean scores for each subscale were calculated; higher scores indicated greater empathy. The total score achieved good internal reliability (Cronbach’s ⍺ = 0.86).

### Procedure

Small groups (between 5 and 10) of participants took part in the study in a large testing room, with individual participants spaced around the room in private computer cubicles. The blinds were drawn, and the same lighting was used for each testing session. Before data collection, participants read the study information sheet and provided written informed consent. Participants were then seated 60 to 65 cm from the screen and completed a 9-point calibration. If the calibration quality was poor, the process was repeated. No participant was asked to complete the calibration more than three times. They then completed both questionnaires via the online survey software, Qualtrics, before starting the social perception task. Participants were instructed to watch the videos in a free viewing task. Each of the 60 trials consisted of a grey background with a black fixation cross presented in the middle of the screen for 10 s^2^ before the 4-s video. The videos began automatically or were manually triggered earlier by the participants, who were instructed to focus on the fixation cross before beginning each video. The videos were presented in random order and occupied the full screen. Participants’ eye movements were recorded throughout the task. At the end of the study, participants were fully debriefed before leaving.

### Data analysis

All statistical analyses were conducted in R (version 1.4.1717) using the lme4 package (Bates et al., 2015). Satterthwaite’s approximate method was used for significance testing (Kuznetsova et al., 2017). The data and analysis code are available on OSF (https://osf.io/dqjpr/?view_only=01bdb55be14148e4ae4121b5f64489fa).

Dynamic areas of interest (AOIs) were drawn around the two agents in each video (see Figure 1) using iMotions. These AOIs were adjusted on a frame-by-frame basis to ensure that all the human figure was captured in each frame. For example, if only the leg of an agent was visible at the beginning of a video, as they entered the scene, the AOI was increased to include their full body, or vice versa. If an agent was holding or began to interact with an object, the object was not included in the agent AOI (objects always formed part of the background AOI throughout the video). Static and dynamic examples of the AOIs are available on the study’s OSF page (https://osf.io/dqjpr/?view_only=01bdb55be14148e4ae4121b5f64489fa). A static AOI was also created for each video capturing the whole scene. Fixation times (sum of time the participants were fixated in an AOI while the stimulus was on the screen) were then exported for all AOIs. Thus, by subtracting the fixation time for both agents from the scene AOI, we could create a measure of time spent looking at the background AOI in each video.

Linear mixed-effect (multilevel) modelling was carried out to assess whether participants spent more time looking at the agent AOIs compared to the background AOIs, and whether looking time differences were moderated by the presence of a social interaction. Thus, modelling focused on a 2 (Stimulus: Interacting = 1 vs. Non-Interacting = 0) × 2(AOI: Agent = 1 vs. Background = 0) design. Working from the null/unconditional model, then adding random then fixed effects, models assessed the impact of individual differences in empathy and autistic traits. Participants were modelled as a random intercept effect; stimulus, AOI, IRI scores and AQ scores as fixed predictors.

## Results

**Figure 2.**
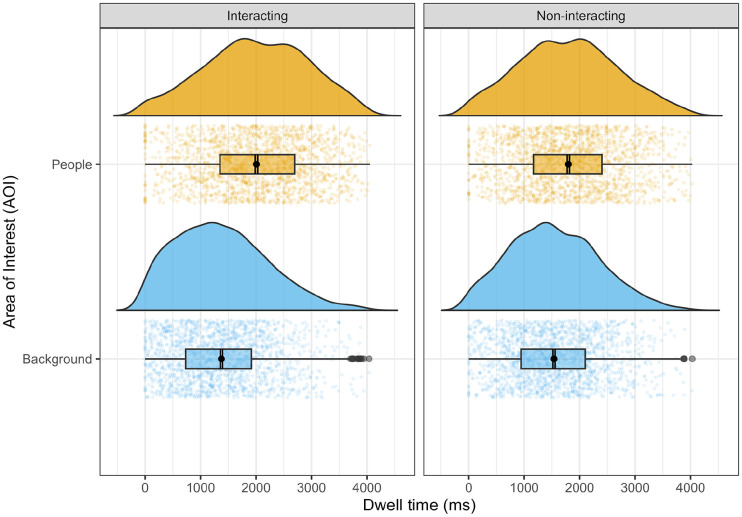
Participants' fixation time (in ms) as a function of stimuli and AOI. *Note*. AOI = areas of interest.

Model 1 included participants as a random intercept with stimulus and AOI as fixed effects (see Table 1 for full details). Despite the similar stimulus presentation time, there was a small main effect of stimulus (*t* = 5.53, *p* < .001, *d* = 0.13), such that participants spent slightly more time fixating at videos depicting interactions versus non-interactions. There was also a significant main effect of AOI (*t* = 21.66, *p* < .001, *d* = 0.52), such that participants spent more time looking at the agents versus the background. Crucially, there was also a significant interaction in the anticipated direction (*t* = −9.09, *p* < .001, *d* = −0.22): participants spent more time looking at the agent AOIs (compared to background AOIs) when viewing social interactions (compared to non-interactions) (see Figure 2).

**Table 1.** Linear mixed-effect model statistics for fixation time without individual differences..

	Fixed effects					
	*b*	*SE*	95% CI	*t*	*p*	*d*
Intercept	1,377.22	22.99	1,332.15, 1,422.29	59.90	<.001	
Stimulus	161.94	29.27	104.57, 219.32	5.53	<.001	0.13
AOI	630.55	29.12	573.47, 687.63	21.66	<.001	0.52
Stimulus × AOI	−373.00	41.05	−453.47, −292.53	−9.09	<.001	−0.22
	Random effects					
	Variance			*SD*		
Participant	5,878			76.67		
	Model fit					
	Marginal			Conditional		
*R* ^2^	.072			.080		

*Note*. Number of total observations = 7,018. Number of participants = 61. Model equation = DT − (1 | participants) + stimulus + AOI + stimulus × AOI. AOI = areas of interest.

**Figure 3.**
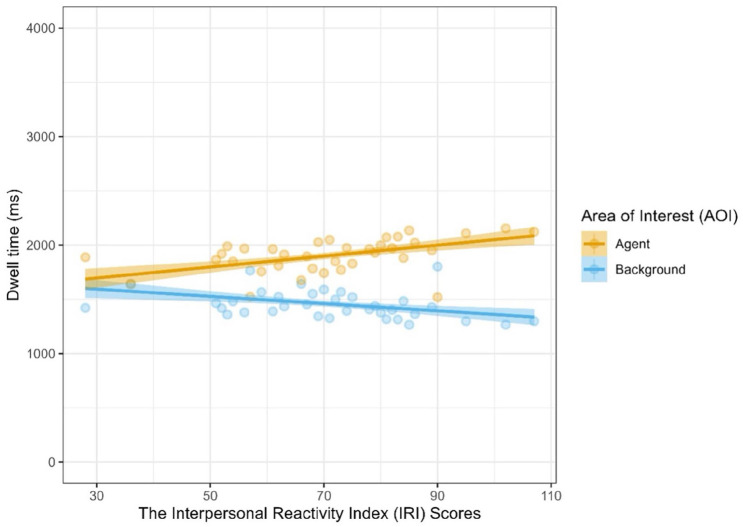
Participants’ fixation time (in ms) as a function of AOI and IRI scores. *Note*. AOI = areas of interest; IRI = Interpersonal Reactivity Index.

**Figure 4.**
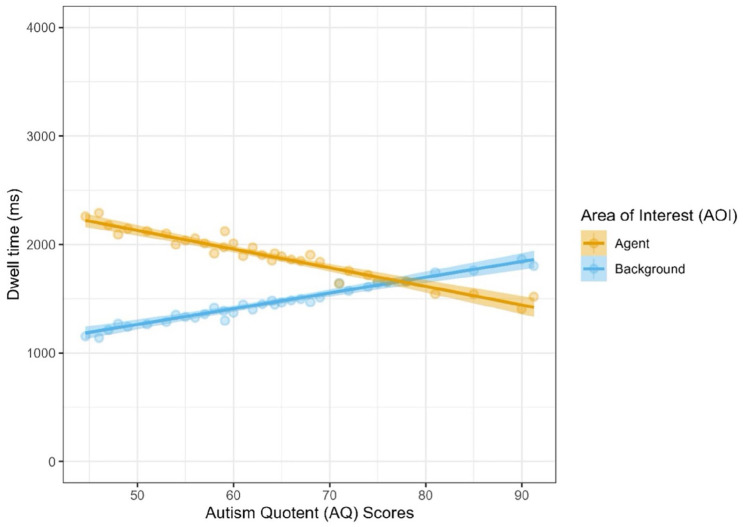
Participants’ fixation time (in ms) as a function of AOI and AQ scores. *Note*. AOI = areas of interest; AQ = Autism Quotient.

The model achieved an overall better fit, however, when individual differences in empathy and autistic traits were added in (Model 1: Marginal *R*^2^ = .072, Conditional *R*^2^ = .080: Model 2: Marginal *R*^2^ = .107, Conditional *R*^2^ = .114; ∆χ^2^(8) = 267.28, *p* < .001). Again, there was a significant main effect of AOI (*t* = 7.39, *p* < .001, *d* = 0.18), but there were also significant interactions between AOI and both IRI (*t* = 4.25, *p* < .001, *d* = 0.10) and AQ scores (*t* = −9.80, *p* < .001, *d* = −0.23). Participants with greater trait empathy spent longer looking at the agent AOIs (see Figure 3) and participants with more autistic traits spent longer looking at the background AOIs. Based on a visual inspection of the model estimates, participants with particularly high autistic traits (AQ-S >76) in our sample appear to have crossed over to spend *more* time looking at the background AOIs than the agent AOIs (see Figure 4). These trait-related effects, however, were not further moderated by whether videos depicted interactions or not (Table 2).

**Table 2.** Linear mixed-effect model statistics for fixation time with individual differences.

	Fixed effects					
	*b*	*SE*	95% CI	*t*	*p*	*D*
Intercept	*796.41*	*192.35*	*419.36*, *1,173.47*	*4.14*	*<.001*	
Stimulus	−130.76	245.24	−611.50, 349.98	−0.53	.594	−0.01
AOI	*1,802.30*	*243.93*	*1,324.13*, *2,280.46*	*7.39*	*<.001*	*0.18*
IRI	−*3.26*	*1.60*	−*6.40*, −*0.12*	−*2.04*	*.042*	−*0.20*
AQ	*12.78*	*2.26*	*8.35*, *17.22*	*5.65*	*<.001*	*0.56*
Stimulus × AOI	276.99	343.21	−395.80, 949.78	0.81	.420	0.02
Stimulus × IRI	1.73	2.05	−2.28, 5.73	0.85	.398	0.02
AOI × IRI	*8.63*	*2.03*	*4.65*, *12.60*	*4.25*	*<.001*	*0.10*
Stimulus × AQ	2.70	2.89	−2.96, 8.36	0.93	.351	0.02
AOI × AQ	−*28.11*	*2.87*	−*33.74*, −*22.49*	−*9.80*	*<.001*	−*0.23*
Stimulus × AOI × IRI	−4.46	2.86	−10.07, 1.15	−1.56	.119	−0.04
Stimulus × AOI × AQ	−5.29	4.05	−13.22, 2.64	−1.31	.191	−0.03
	Random effects					
	Variance			*SD*		
Participant	5,542			74.44		
	Model fit					
	Marginal			Conditional		
*R* ^2^	.107			.114		

Numbers in italics represent significant effects and interactions. Number of total observations = 7,018. Number of participants = 61. Model equation = DT − (1 | participants) + stimulus + AOI + IRI + AQ + stimulus × AOI + stimulus × IRI + AOI × IRI + stimulus × AQ + AOI × AQ + stimulus × AOI × IRI + stimulus × AOI × AQ. AOI = areas of interest; IRI = Interpersonal Reactivity Index; AQ = Autism Quotient.

## Discussion

The present study investigated visual attention as a potential mechanism that might facilitate social interaction perception. Crucially, the study sought to extend existing research by using *dynamic naturalistic* stimuli, thereby examining whether visual attention is still captured preferentially by social interactions even when motion cues – which also drive visual attention – are present. The findings support our first hypothesis; participants spent longer looking at the two agents in the video relative to the background when they were interacting compared to not interacting. In addition, there were also individual differences in the extent to which social information captured attention. There was partial support for our second hypothesis that participants higher in trait empathy would display greater visual attention in response to social interactions (compared to non-interactions), in that participants with higher scores in trait empathy demonstrated an increased social bias (i.e. more time spent looking at the agents in the video and less time looking at the background). Similarly, there was partial support for our final hypothesis that participants lower in autistic traits would display greater visual attention in response to social interactions (compared to non-interactions), such that participants with higher levels of autistic traits were associated with a reduced social bias. Interestingly, the model visualisation suggests a potential cut-off point on the AQ-S where individuals’ preference for looking swapped from the agents to the background (a score of approximately 76–77 in our sample). However, contrary to our expectations, there was no significant interaction between either empathy or autistic traits and whether videos depicted interactions or not, suggesting that these differences in social bias were not moderated by the presence of social interactions.

Our findings support and extend the small body of research investigating the visual processing of social interactions (Chevallier et al., 2015; Fratino et al., 2022; Papeo & Abassi, 2019; Skripkauskaite et al., 2022; Vestner et al., 2019). Specifically, we replicate an earlier finding that social interactions capture visual attention to a greater extent than non-interactions in naturalistic stimuli (Skripkauskaite et al., 2022), but extend this finding by demonstrating this in *dynamic videos* that may provide both additional cues to interaction (e.g. coordinated or reciprocal actions) but also greater distraction (e.g. ‘low level’ motion cues capturing attention; the need to track two individuals’ goals) in the non-interaction condition. We also replicate and extend findings from the only study to date to assess visual attention to social (faces) versus non-social (objects) AOIs in naturalistic dynamic stimuli (Chevallier et al., 2015). In their study, participants demonstrated greater looking to faces versus objects, and thus our findings (a) confirm that whole bodies also capture visual attention to a greater extent than background objects in naturalistic dynamic stimuli and (b) extend this by demonstrating this effect to be moderated by the presence of a social interaction. We note that Chevallier et al. (2015) did include interaction versus non-interaction conditions in their task. However, as this factor was not included in their analysis, it is not possible to draw any conclusions regarding social interaction perception from their study.

Why might individuals spend more time looking at two agents when they are interacting as opposed to when they are engaged in pursuing separate goals in these complex dynamic scenes? As discussed in prior work (Skripkauskaite et al., 2022), social interactions provide unique information about the social context and the relationship between people in a scene. Thus, social interactions give viewers information by which to predict future *social* events that viewing individual actions rarely provides. In addition, and as a potential explanation for why we observed greater visual attention to interacting people in *dynamic stimuli*, as the interaction unfolds over time individuals may spend more time looking back and forth between the agents (compared to non-interacting individuals). Gestures and gaze cues directed towards the other person may also drive attention back and forth between interactants rather than towards objects or background elements. Future research may wish to investigate these ideas using scan path analysis. Lastly, people find interactive scenes – even when they are low-emotive everyday scenes – to be more interesting and more positive than non-interactive scenes, even when these scenes are visually similar, matched for motion energy and include the same agents performing similar actions. In our view, this is a feature of social interactions rather than a design flaw. Social interactions may draw visual attention *because* humans are inherently social and pursue understanding interactions and relationships even when doing so serves no obvious purpose for the observer.

Conversely, instead of considering how interactions might increase social attention, one might instead ask why individuals spend more time looking at background elements in non-interactive (compared to interactive) scenes. Our findings don’t necessarily show that attention to the background is increased for non-interactive scenes, as the bias to look at social information (i.e. the people) is still present, but weaker, for non-interactive scenes. One possibility is that the same sort of attentional cues that drive attention towards social information in interactions is driving attention to objects and background elements in non-interactive scenes. Observers may spend a bit more time looking at objects in such scenes because the people they are observing are interacting with and looking towards objects rather than other people in the scene. This suggestion, that gaze and facing direction cues may increase attention to people in interactive scenes and to objects in non-interactive scenes, is congruent with at least some aspects of previous work from Vestner and colleagues.

This work (Flavell et al., 2022; Vestner, Gray, et al., 2021, 2022; Vestner, Over, et al., 2022) has postulated that the observed visual attention bias towards ‘social interactions’ is in fact driven by ‘low level’ or non-specific attentional cues to do with the visual properties of the stimuli. In this work, static images of agents are shown facing towards, or away from, one another. They propose that when two agents are facing each other, the ‘visually interesting’ side of the body (the front) is visually closer together (compared to when the agents face away from one another) and that it is this orientation of interesting shapes ‘pointing’ to one another that is driving visual attention, so-called attentional cuing. Objects that spatially cue attention towards each other create a ‘hot spot’ that captures and holds attention, regardless of whether those objects are socially relevant or not. To support this idea, they have replicated greater attention to facing than non-facing objects for objects that spatially direct attention (i.e. for objects for which there is a Posner cuing effect; Vestner, Over, et al., 2022). While it was not the intention of the current study to directly examine this theory, our findings may offer some interesting insight. Firstly, our stimuli are deliberately extremely variable within conditions (social interaction vs. non-interaction), such that while agents may be facing each other in some social interactions, in others, they may not. In some social interactions, they may be talking with one another, in others they may be jointly attending to an object. Crucially, despite this deliberate *within* condition variability, the scenarios were tightly matched *across* conditions, such that each specific scenario was as similar as possible between the social interaction and non-interaction version. This included matching as closely as possible proximity, facing direction, objects, movement and even eye-gaze when possible. The intention was that by varying these typical interaction cues *within* conditions, and controlling for them *across* conditions, we would be able to generalise our findings to a more abstract and holistic idea of social interaction in the truest (and messiest) sense. Secondly, simply by introducing motion into the stimuli, these various cues to social interaction often unfolded and changed throughout a video, adding an additional layer of complexity to the stimuli (compared to previous studies). Consequently, it is unlikely that simple ‘low level’ cues can fully explain visual attention in our dynamic naturalistic stimuli. However, because the current study did not require participants to engage in any task associated with the stimuli, we cannot speak to how participants interpreted or processed the scenarios. We therefore tentatively suggest that the attentional bias observed in the current study is at least partially driven by higher order processing of the stimuli, but that future studies will be required to fully confirm this.

Our findings are also broadly in line with previous research investigating individual differences in visual attention. While Villani et al. (2015) found that individuals with greater empathic concern (a subscale of the IRI) were faster (fewer fixations prior) to look to the face, they found no effect of any IRI subscale on looking time. In contrast, the present study found that individuals higher in trait empathy (represented by the whole IRI scale) spent longer looking at the agents. The findings across studies are broadly compatible – individuals with higher empathy show a stronger attentional bias towards social information – and differences between studies are potentially explained by methodological differences. Villani et al. presented static images of artwork for 15 s, while the current study presented naturalistic dynamic videos for 4 s. First, time-to-first-fixation or similar metrics are not relevant in stimuli that unfold over time and thus were not calculated in the current study. Secondly, the longer presentation time used by Villani and colleagues may have resulted in general processing of the image towards the end, potentially masking possible biases in looking time that might have been present during early processing. Chevallier et al. (2015)’s primary interest was to examine differences in visual processing of social information between autistic and non-autistic individuals. Indeed, and in line with our findings, they found that autistic individuals spent less time looking at social AOIs and more time looking at object AOIs. Thus, our results confirm that individuals with higher levels of autistic traits also display differences in social information processing in naturalistic dynamic stimuli. We note, however, that our individual difference findings were not moderated by the presence of social interactions. This may reflect a genuine finding, suggesting that individual differences could explain a significant portion of variance in our main finding. However, we suggest this is unlikely due to the within subject design. Our study was powered for a three-way rather than a four-way interaction, and so it is possible that we may not have had enough power to detect higher order effects in our more complex model incorporating individual differences. Future work, perhaps manipulating social scenes to include stimuli that induce empathic responses, could delve more deeply into individual differences that might specifically influence attention specifically to social interactions.

Although it is possible that we did not have enough power to detect a four-way interaction, we highlight several strengths of the present study. First, the study recruited a good sample size (*N* = 85, eye tracking observations = 7,018) and utilised mixed-effect modelling to control for random variation across participants. Second, the study used naturalistic dynamic videos and, as such, provides novel insight into the processing of social interactions in more ecologically valid stimuli. Third, the stimulus set, although naturalistic, is tightly controlled such that important factors such as proximity between agents, props, actions, facing direction and level of motion are equivalent (or as similar as possible) across specific interaction and non-interaction contexts. Fourth, although our stimuli are as tightly controlled as possible *across* conditions (forming interaction and non-interaction pairs) within conditions there is a wide range in these variables, thus any findings across the interaction category cannot be related to specific low-level visual features, but rather the intended contrast of the presence or absence of a social interaction. Fifth, although the mean age (30.78) of the current study is close to the typical student populations utilised in many psychological experiments, our age range (19–84) incorporated a much wider proportion of the population and achieved a roughly 50/50 sex split, thereby increasing the generalisability and inclusivity of the study.

In conclusion, the present study investigated visual attention as a potential mechanism through which individuals may process social interactions. Importantly, the current study used naturalistic dynamic stimuli and therefore provides novel insights into social interaction processing. The study found preferential processing of people in social interactions versus non-interactions, measured via longer looking times. There were also individual differences in this processing, such that individuals higher in trait empathy spent longer looking at people relative to the background, while individuals higher in autistic traits spent longer looking at the background. Future studies may wish to explore whether these longer looking times are driven by continual looking back and forth between interactants as the social interaction unfolds over time.

## Notes

1. We note that the pre-registration for this study included the collection of facial movement data, which was included as an additional, exploratory mechanism for social interaction perception. The data, rationale and results of the facial data are not included in the manuscript due to data issues rendering half the data unanalysable and the remaining data of very limited use. For full transparency, a brief report of this data is available on the study’s OSF page (https://osf.io/dqjpr/?view_only=01bdb55be14148e4ae4121b5f64489fa).

2. This was due to an iMotions programming limitation of a minimum presentation time of 10 s.
